# Benefits of Organo‐Aqueous Binary Solvents for Redox Supercapacitors Based on Polyoxometalates

**DOI:** 10.1002/celc.202000639

**Published:** 2020-06-10

**Authors:** Sonia Dsoke, Qamar Abbas

**Affiliations:** ^1^ Helmholtz Institute Ulm for Electrochemical Energy Storage (HIU) Helmholtzstraße 11 89081 Ulm Germany; ^2^ Institute for Applied Materials Karlsruhe Institute of Technology Hermann-von-Helmholtz-Platz 1 Eggenstein-Leopoldshafen Germany; ^3^ Institute for Chemistry and Technology of Materials Graz University of Technology Stremayrgasse 9 8010 Graz Austria

**Keywords:** polyoxometalates, heteropolytungstate, redox supercapacitors, electrolyte, water/dimethyl sulfoxide mixtures

## Abstract

A novel redox electrolyte is proposed based on organo‐aqueous solvent and a polyoxometalate (POM) redox moiety. The presence of dimethyl sulfoxide (DMSO) plays multiple roles in this system. Firstly, it enhances the cathodic electrochemical stability window by shifting the H_2_ evolution to lower potentials with respect to pure aqueous systems; secondly, it improves the reversibility of the redox reaction of the PW_12_O_40_^3−^ anion at low potentials. The presence of DMSO suppresses the Al corrosion, thus enabling the use of this metal as the current collector. An activated carbon‐based supercapacitor is investigated in 1 M LiNO_3_/10 mM H_3_PW_12_O_40_ in a mixed DMSO/H_2_O solvent and compared with a POM‐free electrolyte. In the presence of POMs, the device achieves better stability under floating conditions at 1.8 V. At 1 kW kg^−1^, it delivers a specific energy of 8 Wh kg^−1^ vs. 4.5 Wh kg^−1^ delivered from the POM‐free device. The H_2_ evolution is further shifted by the POMs adsorbed on the activated carbon, which is one reason for the improved stability. The POM‐containing cell demonstrates a mitigated self‐discharge, owing to strong POMs adsorption into the carbon pores.

## Graphical abstract

**Present and correct**: The presence of dimethyl sulfoxide (DMSO) in water can drastically improve the properties of an electrochemical system: the hydrogen evolution shifts to lower potential due to the strong interaction of DMSO with water. DMSO suppresses the corrosion of aluminum and stabilizes the reduced form of polyoxometalates (POMs). Organo‐aqueous electrolytes based on DMSO, H_2_O and POMs are thus applied in a supercapacitor device.

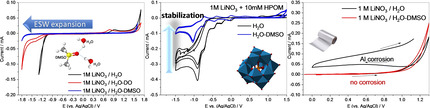

## 1 Introduction

Energy, in the form of electricity, is required in all aspects of life, at home and in the office and to perform our daily tasks, like using smartphones, tablets, computers or other electronic devices. Depending on the specific application, either high energy or high power is desired. Devices, which can provide high energy are based on battery materials, where faradaic (and generally slow) reactions occur.^1^ On the other side, high power can be achieved by the physical storage of energy via electrical double layer formation.^2^ These two are the specific cases of batteries and supercapacitors, respectively. It is possible to find other systems whose characteristics lie in between batteries and supercapacitors, in the form of “hybrid devices.”^3^ The hybridization aims to increase the power of a battery or to enhance the energy density of a supercapacitor. One method to achieve hybridization is to mix two different types of materials belonging to the category of batteries and supercapacitors in one single electrode^4^. Another strategy is to adopt an “asymmetric” configuration, where one electrode is a pure capacitor‐type and the other one a pure battery‐type.^4^ Both strategies focus on solid‐state electrode materials.

Another intriguing method is the introduction of a faradaic material as a moiety dissolved in the electrolyte.^5^, ^6^, ^7^, ^8^ With this approach, the electrolyte contains an active faradaic material, which reacts at the surface of the capacitive‐type electrode material, thus providing the additional capacity. The capacitive‐type material is usually activated carbon, which can offer relatively high capacitance owing to its extremely high surface area (about 1500–2000 m^2^ g^−1^).^9^ Electrolytes for supercapacitors can be mainly categorized in organic and aqueous‐based. Aqueous electrolytes offer the advantage of having high ionic conductivity and low viscosity, which enhances the power capability.^10^ Also, aqueous systems are non‐flammable, can be handled without special environmental conditions, and are relatively cheap. Their main limitation is the narrow electrochemical stability window (ESW) limited by the water‐splitting reactions (H_2_ and O_2_ evolution). Organic electrolytes, on the other hand, have safety and cost concerns, they need to be handled under an Argon atmosphere (due to the strictly anhydrous condition requirements), but they have the advantage to exploit a larger ESW, which turns in higher energy density than the aqueous ones.^11^, ^12^ Besides aqueous and organic electrolytes, highly viscous systems, such as ionic liquid, gel, and solid systems (polymeric or ceramic), can be used in supercapacitors, with drawbacks in ionic conductivities.^12^

To date, the reported redox‐active electrolytes are mainly aqueous‐based and various redox species are, so far, applied in redox electrolytes for supercapacitors. Bromine and iodine, highly soluble species in aqueous media, which reacts at the positive electrode, were the first moieties used as redox electrolytes.^13^ In later works, another redox specie, the vanadyl sulfate, which stores the capacity at the negative electrode, has also been used.^14^ Afterward, many other soluble redox species were investigated, which include hydroquinone,^15^ inorganic and organometallic complexes (ferricyanide, metal bipyridine complexes, etc.),^16^ and organic molecules (phenylenediamine, viologens, quinones, etc.).^17^ Several exhaustive reviews resume the performance of these redox electrolytes in detail.^5^, ^6^, ^7^, ^8^, ^18^ Considering other promising redox moieties, polyoxometalates (POMs) have attracted our attention for several reasons: (i) the electrochemistry of POMs (especially of the Keggin‐type class) is well known;^19^ (ii) POMs are very stable and have the ability undergoing reversible multi‐electron transfer, which is an excellent pre‐condition for energy storage capability;^20^ (iii) they are widely used in electrochemical energy storage systems, like in Li‐ and Na‐ion batteries,^21^ in supercapacitors^22^ and as well as in fuel cells systems.^23^ In all the examples mentioned above, POMs are used in their solid‐state form as electrodes. Due to their good solubility, their potentiality to provide energy in the liquid form has been explored in redox‐flow cells.^24^

In the field of supercapacitors, Nuckoswka et al. provided a proof of concept of a dual redox electrolyte, where a polyoxometalate serves as a redox specie at the negative electrode, and hydroquinone is confined as redox‐active moiety at the positive electrode.^25^ In their work, an aqueous‐based electrolyte is used, which limits the operational cell potential difference to 0.8 V. Higher potential difference in water‐based electrolytes can results in fast degradation due to the evolution of H_2_ and O_2_. The restriction of the potential difference is also due to the irreversible redox reaction of Keggin‐type polyoxometalates at low potentials in an aqueous medium. However, it is proved that the presence of an additional organic solvent can stabilize the reduction process of polyoxometalates at low potentials.^26^, ^27^ The organic solvent can play a role in coordinating the polyoxometalate and as well as influencing the redox potential of the multistep reactions. Stimulated by these findings, herein we aim to study if the addition of an organic solvent to water can stabilize the POM moiety and, at the same time, enlarge the ESW. The focus is on mixtures of water with DMSO and 1,4‐Dioxane. Both solvents are miscible with water and compatible with Keggin‐type POMs.

## 2 Results and Discussion

**Figure 1:**
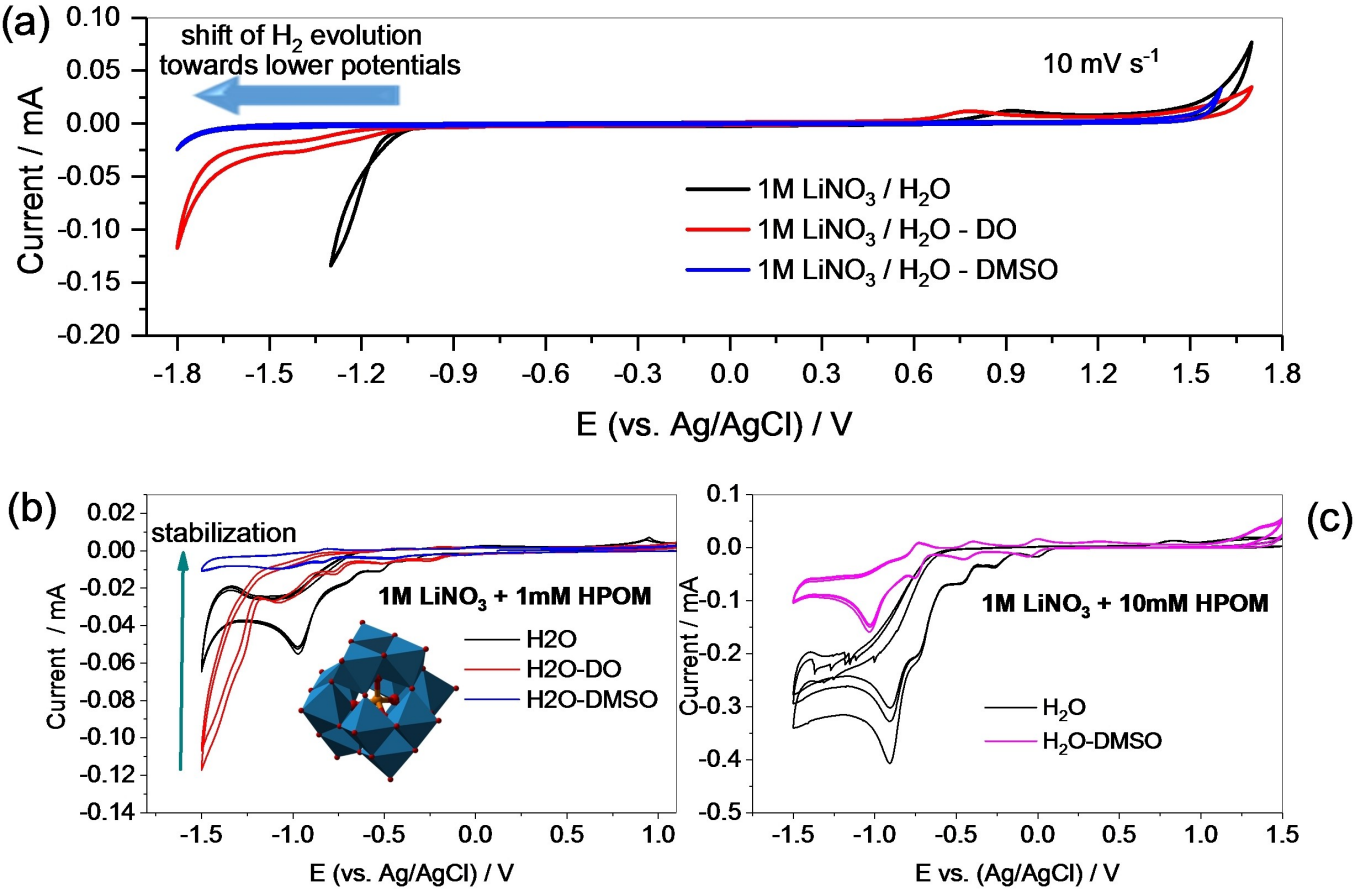
Cyclic voltammograms at 10 mV s^−1^ on glassy carbon electrode of a) 1 M LiNO_3_ salt dissolved in pure H_2_O and organo‐aqueous solvent in the volume ratio 1 : 1; b) the same electrolytes with the addition of 1 mM H_3_PW_12_O_40_; c) the comparison of 10 mM H_3_PW_12_O_40_ in pure water and DMSO‐H_2_O.

Figure 1a shows cyclic voltammetry curves performed on Glassy Carbon electrodes of electrolytes based on 1 M LiNO_3_ in different solvents: pure water, a mixture of water and 1,4‐dioxane (DO), and a mixture of water and DMSO in volume ratio 1 : 1. The presence of the organic solvent significantly shifts the H_2_ evolution to lower potentials, thus enlarging the electrochemical stability window (ESW). Hydrogen evolution starts at −1 V vs. Ag/AgCl in the pure water‐based electrolyte, while it starts at −1.7 V in the presence of DMSO. In the electrolyte based on DO‐H_2_O some decomposition already starts at −1.1 V vs. Ag/AgCl, followed by H_2_ evolution, which starts at about −1.6 V vs. Ag/AgCl. This effect can be attributed to the modification of the water structure induced by the presence of the second solvent. DMSO causes the breaking of hydrogen bonds between water molecules because the interaction DMSO‐H_2_O is stronger than the interaction H_2_O‐H_2_O.^28^ Water structure modification occurs, in a similar fashion, in mixtures DO‐H_2_O.^29^ The presence of organic solvents in water can act in a similar way as the “water in salt electrolyte” (WISE) concept.^30^ The main principle of WISE is to employ all water molecules in the solvation of the ions so that no “free” H_2_O will be present (suppression of H_2_ and O_2_ evolution).

Figure 1a & b shows the effect of the addition of H_3_PW_12_O_40_ heteropolyacid (HPOM) to the 1 M LiNO_3_‐based electrolytes. In agreement with previous electrochemical studies, HPOM in the pure aqueous system undergoes an irreversible redox reaction at 0.9 V vs. Ag/AgCl, as displayed in Figure 1b & c (black curves).^27^ In mixed solvents, this reaction stabilizes and becomes more reversible. Figure 1b shows the CV of 1 mM HPOM in different electrolytes and demonstrates that the best stabilization is obtained with mixed solvents DMSO‐H_2_O. Therefore, DMSO‐H_2_O mixed solvent was selected for supercapacitor application in next sections. In the experiment of Figure 1c, the concentration of polyoxometalate is increased to 10 mM. While, in pure aqueous electrolytes, it is not possible to observe the returning oxidation peaks, these peaks become visible in mixed organo‐aqueous electrolytes (the zoom on the redox peaks is shown in the ESI file, S1). The addition of DMSO induces an increase in the pH due to its strong interaction with water hydrogen bonds. On the other side, the addition of HPOM, decreases the pH, as it is expected due to its superacidic nature. The conductivity is strongly affected by the solvent but is only slightly dependent on the presence of HPOM (Table 1).

**Table 1:** pH and conductivities of selected electrolytes.

Electrolyte	pH	Conductivity [mS cm^−1^]
1 M LiNO_3_/H_2_O	5.69	65.46
1 M LiNO_3_/DMSO‐H_2_O	6.64	17.28
1 M LiNO_3_+1 mM H_3_PW_12_O_40_/H_2_O	2.0	64.25
1 M LiNO_3_+1 mM H_3_PW_12_O_40_/DMSO‐H_2_O	3.0	18.16
1 M LiNO_3_+10 mM H_3_PW_12_O_40_/DMSO‐H_2_O	1.75	19.01

**Figure 2:**
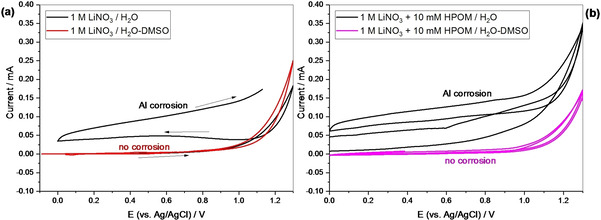
Cyclic voltammograms at 10 mV s^−1^ on an Aluminum foil working electrode. a) Polyoxometalate‐free electrolytes and b) electrolytes containing 10 mM H_3_PW_12_O_40_.

One issue of aqueous‐based electrolytes is that they are incompatible with the Aluminum current collector, which is subjected to corrosion (i. e., removal of the protective Al_2_O_3_ layer).^31^ This fact implies that other metal foils, like stainless steel, have to be used as current collectors in the final supercapacitor device. However, due to its low density, easy processability, and high electronic conductivity, Aluminum is a preferable material in the battery and supercapacitor fields,^32^, ^33^ and it is commonly used with organic electrolytes, where the corrosion is not relevant. Strategies to suppress Aluminum corrosion in aqueous systems are of great importance to decrease the cost of the device and improve the performance.^31^, ^34^ Figure 2 shows that the corrosion of aluminum is suppressed with mixed DMSO‐H_2_O electrolytes. Consecutive cyclic voltammograms scanned from 0 to 1.3 V vs. Ag/AgCl show, in the pure water‐based electrolyte, a current increase with progressive cycling, a signature of continuous corrosion. This phenomenon is not visible in the presence of DMSO. This result is of high significance because it allows using Aluminum as a current collector for the AC‐based electrodes.

**Figure 3:**
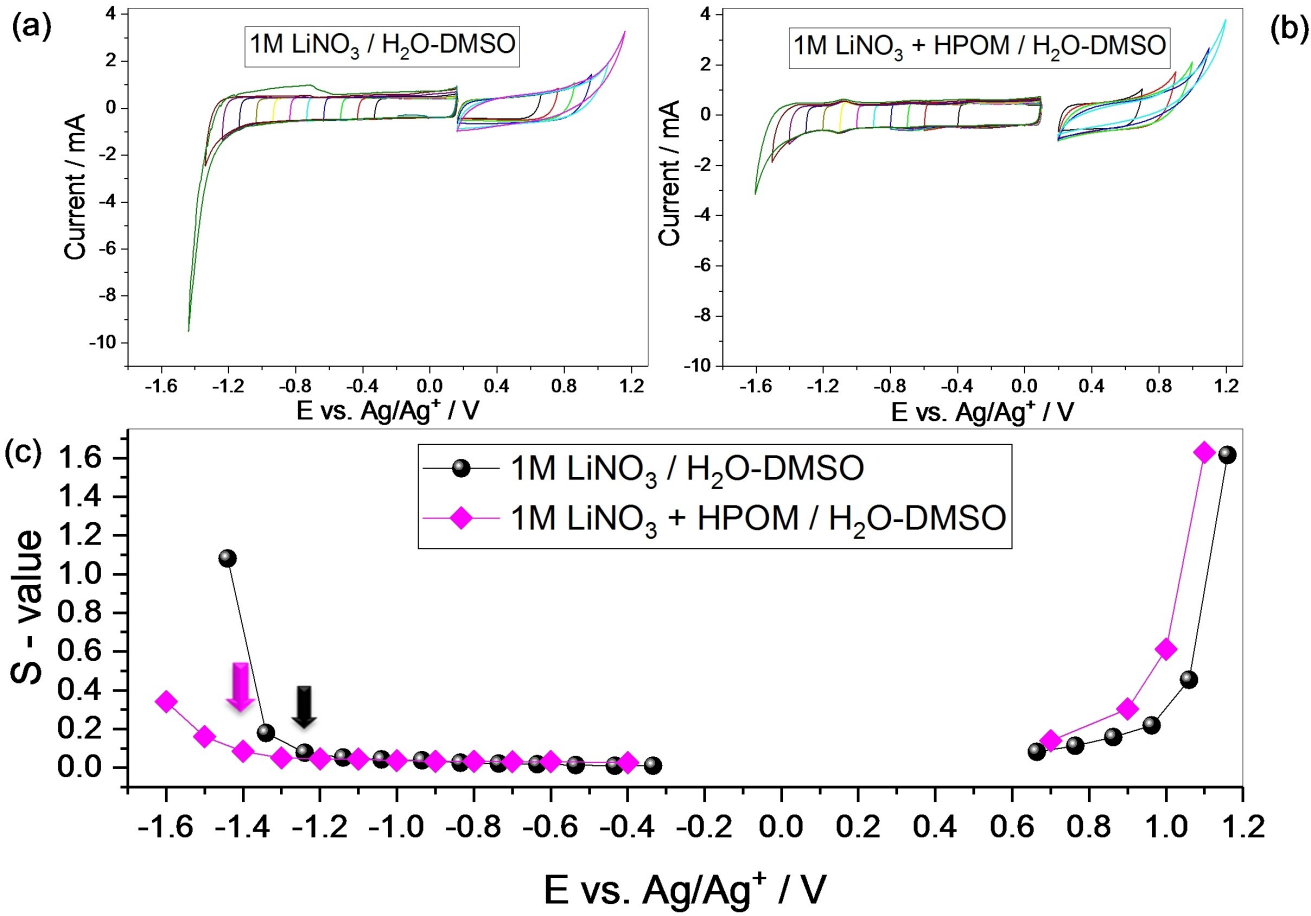
Potential opening experiment on activated carbon electrodes in a) 1 M LiNO_3_/H_2_O‐DMSO and b) 1 M LiNO_3_+10 mM HPOM/H_2_O‐DMSO. c) S‐value versus potential.

Even if Figure 1 gives an indication about the ESW of the electrolyte, this is done on low‐surface electrode material (i. e. glassy carbon). The “realistic” stability window needs to be evaluated with the real electrode substrate, *i. e*. based on activated carbon. Figure 3 shows the potential opening experiment conducted in half‐cell configuration, performed in cathodic and anodic directions (Figure 3a and b). In order to evaluate the ESW electrode/electrolyte, the S‐value (S=Q_charge_/Q_discharge_−1) is calculated and reported as a function of the opening potential.^35^ The S‐value represents the irreversible capacity fraction and gives an indication about the maximum positive and negative potentials, which can then be applied in the full two‐electrode cell. In the positive direction (anodic polarization), the S‐value is slightly lower for the POM‐free electrolyte. In the negative direction (cathodic polarization, where hydrogen adsorption takes place) the stability limit is sensibly wider with the electrolyte containing POMs as indicated by the arrows (Figure 3c).

**Figure 4:**
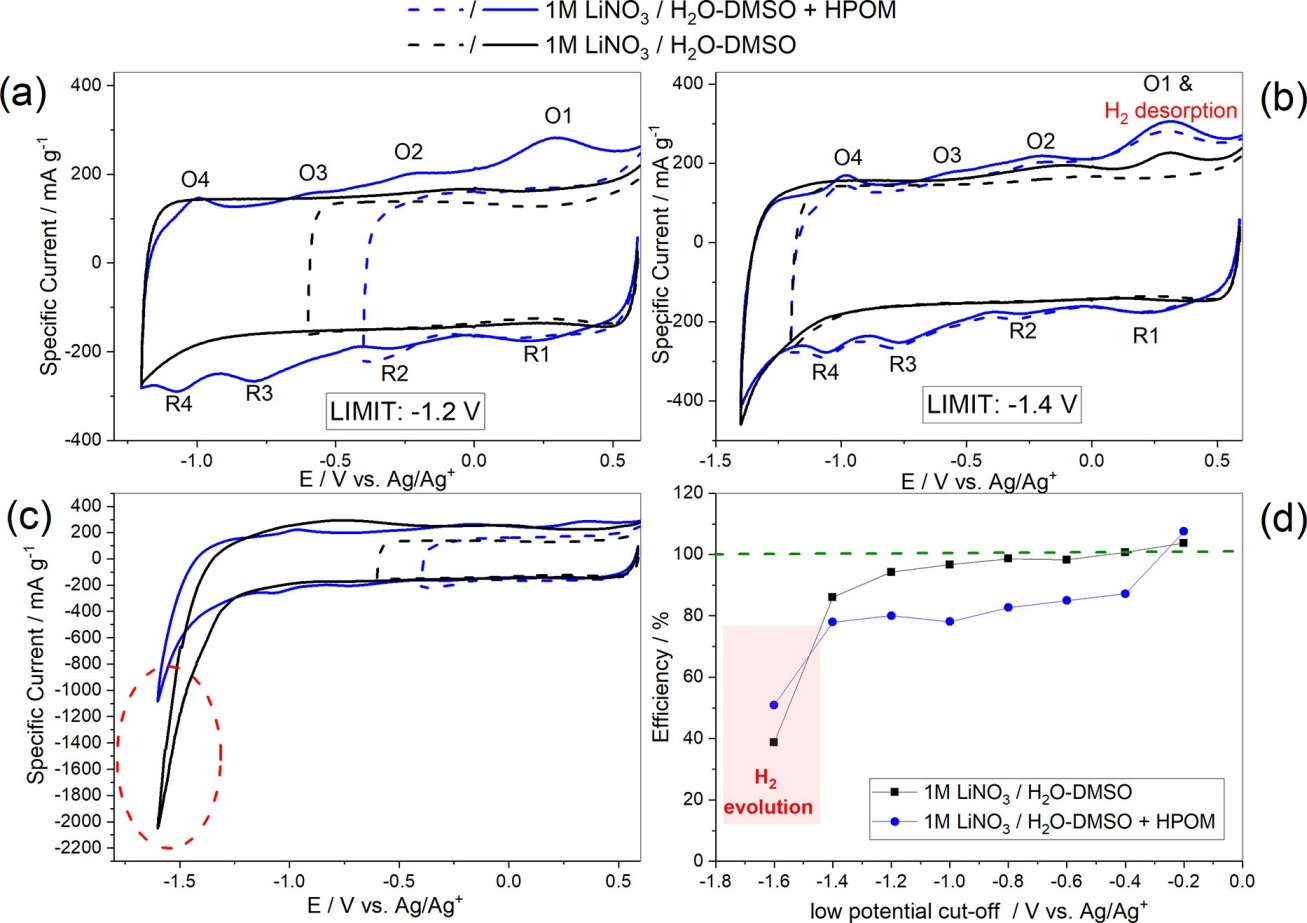
Comparison of cyclic voltammograms (2 mV s^−1^) in three‐electrode setup on AC electrode using 1 M LiNO_3_/H_2_O‐DMSO (black curve) and 1 M LiNO_3_/H_2_O‐DMSO+HPOM (blue curve) by step‐wise cut off potential down to −1.2 V (a), −1.4 V (b), 1.6 V (c). Coulombic efficiency comparison of cells in two electrolytes calculated from CV at different potential steps (d).

**Figure 5:**
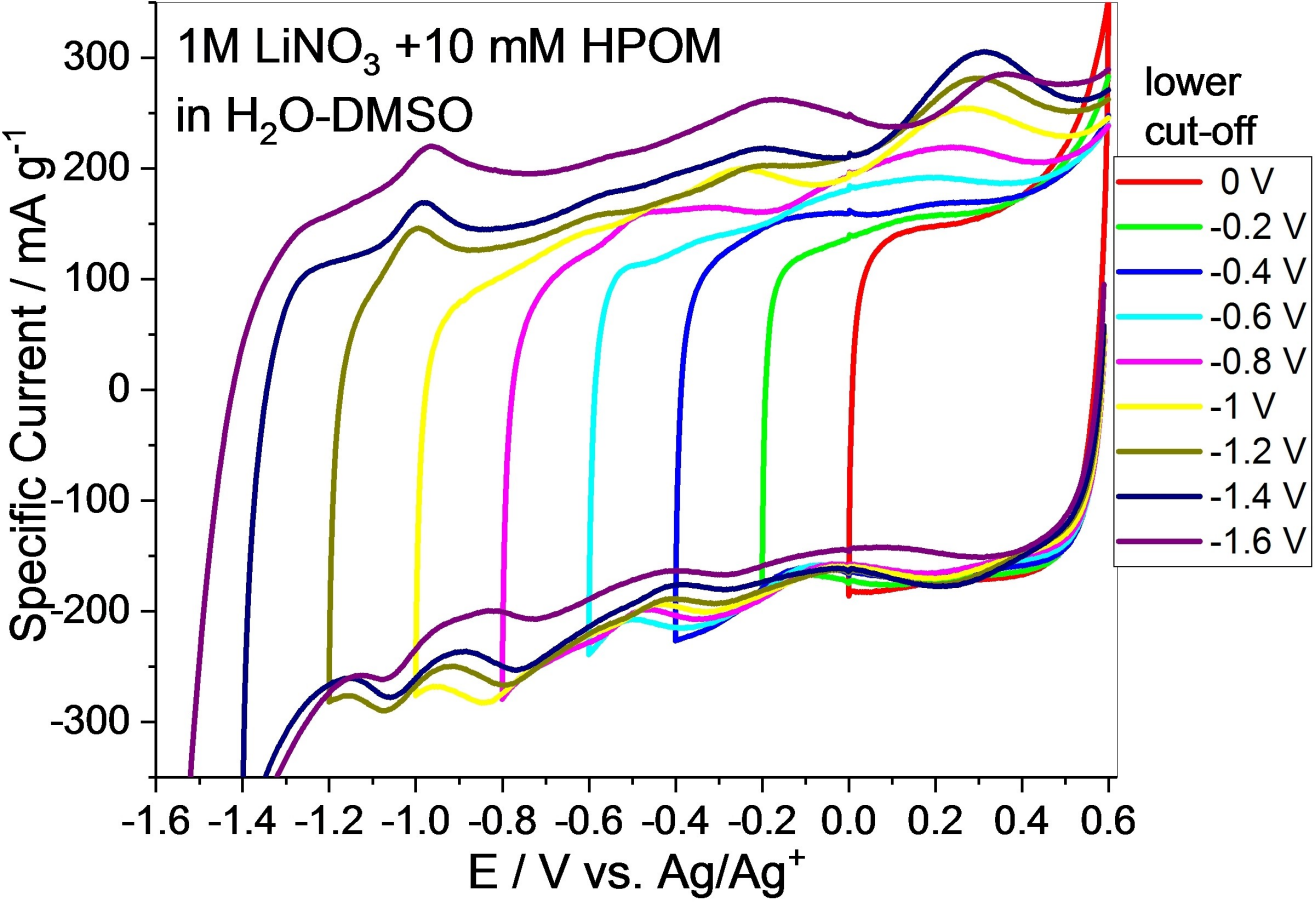
Cyclic voltammograms in the three‐electrode setup on the AC working electrode at 2 mV s^−1^ in 1 M LiNO_3_/H_2_O‐DMSO+HPOM down to various cut‐off potential.

As reported in the literature for aqueous‐based electrolytes, hydrogen can be reversibly adsorbed on porous carbon electrodes and even contribute to the reversible capacity.^36^ Understanding the extent of hydrogen evolution and hydrogen adsorption/desorption on the AC electrode helps to elucidate the storage properties and clarify any degradation mechanism. Figures 4a, b, and c show the comparison of CVs in 1 M LiNO_3_/H_2_O‐DMSO+HPOM (blue curve) and 1 M LiNO_3_/H_2_O‐DMSO (black curve) at different cut‐off potentials. For the CV of the system with POMs, oxidation and reduction peaks characteristics of fully reversible redox reactions appear at specific potential values where each oxidation peak (O) corresponds to a reduction peak (R). In the curve of the cell with a neat LiNO_3_ electrolyte, no redox peak appears down to −1.2 V (Figure 4a). The peak related with hydrogen desorption^36^ (at 0.29 V vs Ag/Ag^+^) is absent in neat LiNO_3_ electrolyte down to −1.2 V vs Ag/Ag^+^. On the other hand, for the cell with 1 M LiNO_3_/H_2_O‐DMSO+HPOM, the reversible redox behavior of the POMs is initially absent (also see Figure 5) and starts to appear when the potential is scanned down to more negative potentials, indicating that the adsorption of POMs takes place under polarization effect and the redox behavior gradually increases when more of these moieties are adsorbed at the AC. The redox reactions are not initially visible, which confirms that, at first, charges are accumulated at the electric double‐layer (EDL). In addition, slightly higher specific current at low potential (Figure 4a, dashed curves), in the system with POMs than in neat LiNO_3_, could be due to the presence of freely available mobile H^+^ species, which favorably participate in EDL formation.

However, the hydrogen desorption potential (0.29 V) coincides with the potential of O1 peak, which is also located at around 0.29 V vs. Ag/Ag^+^ (Figure 4b) and rest of oxidation and reduction peaks become more visible down to −1.4 V, confirming the assumption of strong adsorption of POMs as shown in Figure 5. Obviously, upon progressively scanning the electrode to a lower potential limit, the peaks start to appear, and O1 grows and shifts to slightly higher potential (Figure 5). However, while the current related to O1 increases, the current related to R1 does not change, indicating the additional oxidation of adsorbed hydrogen besides the reoxidation of the product reduced in R1. Further potential decrease down to −1.6 V vs. Ag/Ag^+^ results in hydrogen evolution, which is higher in the case of 1 M LiNO_3_/H_2_O‐DMSO than in 1 M LiNO_3_/H_2_O‐DMSO+HPOM (Figure 4c). This can be explained by enhanced overpotential for hydrogen evolution at the AC owing to the favorable adsorption of POMs, which modifies the hydrogen evolution potential.^37^ Even if less hydrogen is evolved in 1 M LiNO_3_/H_2_O‐DMSO+HPOM, a part of it might be aided by the protons which come from POMs (not only from the free water), and its effects on the aging of the full cell will be discussed later. POMs are superacids materials, and they have acidic protons available in the structure. The influence of faradaic reactions of POMs is confirmed by a lower efficiency of the system containing 1 M LiNO_3_/H_2_O‐DMSO+HPOM with respect to the one without POMs (pure double‐layer always results in higher efficiency due to physical charge storage).

**Figure 6:**
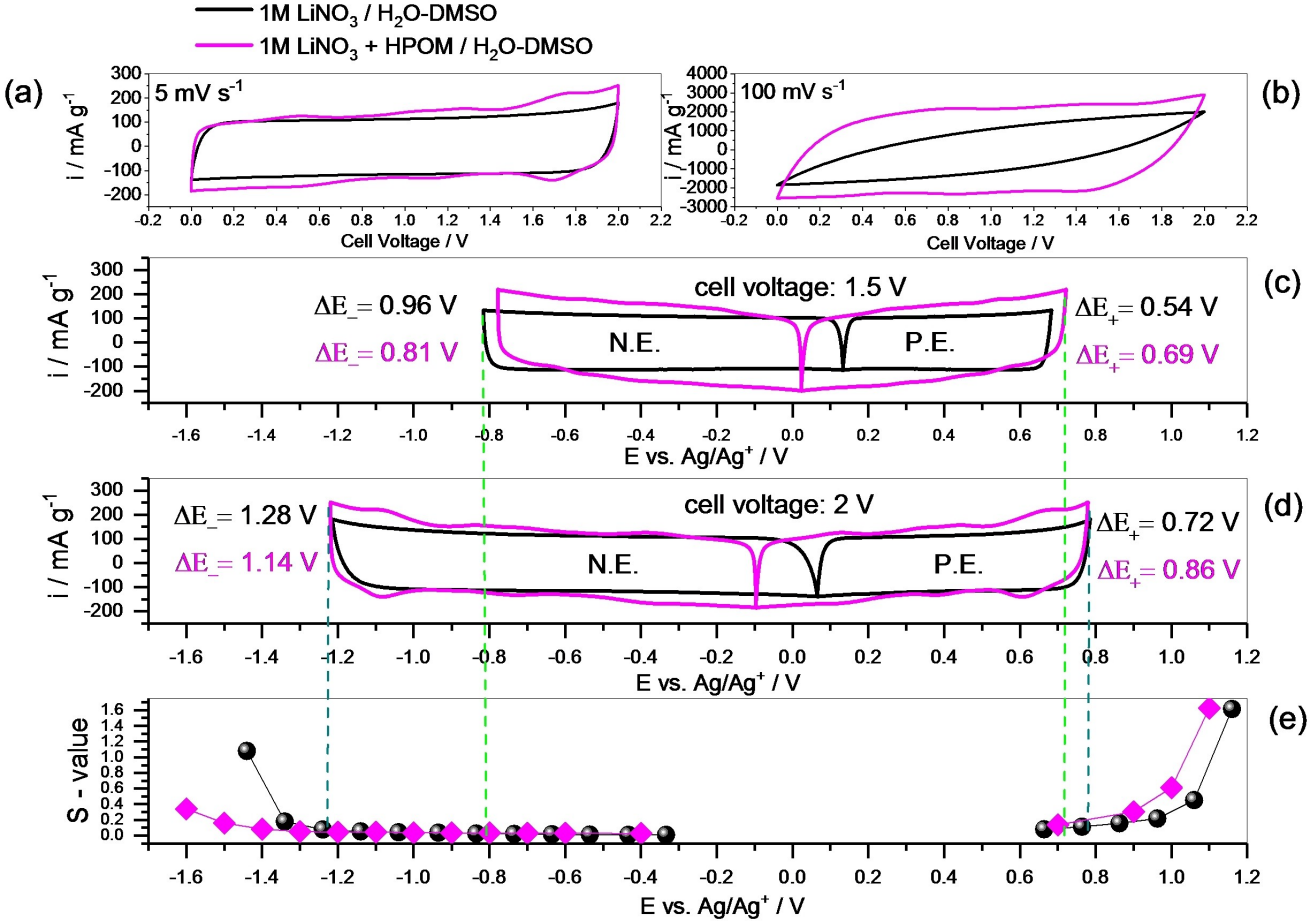
Cyclic voltammetry profiles at 5 mV s^−1^ (a) and 100 mV s^−1^ (b) of AC//AC supercapacitors with a mass ratio of 2 : 1 (positive/negative electrode) and selected electrolytes. Potential swing of positive and negative electrodes at a cell voltage of 1.5 V (c) and at the cell voltage of 2 V (d). Correlation with the S‐values obtained in the half cells (e). (P.E. : positive electrode, N.E. . negative electrode).

**Scheme 1:**
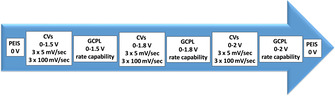
The experimental protocol used in AC/AC full cells.

AC/AC full cells were assembled with a mass ratio between the positive and negative electrodes of 2 : 1, and the cells were evaluated in different potential windows (1.5 V, 1.8 V, and 2 V). With this ratio, the potentials of positive and negative electrodes remain inside the electrochemical stability window (Figure 6) up to the cell voltage of 2 V. The cells were subsequently subjected to PEIS, CV, and GCPL at different cell voltages, as explained in the experimental section (Scheme 1). The CV at low (5 mV sec.^−1^) and high (100 mV sec.^−1^) scan rates at the cell voltage 2 V are compared in Figure 6a and b. A comparison with CV recorded until the cell voltage of 1.5 V is reported in the ESI file, S3. The redox peaks of the HPOM on the AC electrode are still visible but broader respect to what observed on GC electrode surface (compare Figure 1c and S3). The broadening of the redox peaks gives rise to a kind of pseudocapacitive‐like behavior.^38^ The higher specific current, in the presence of HPOM, is an indication of enhanced charge storage on the activated carbon. Notably, when the cell without HPOM is cycled between 0 and 2 V, substantial deformation of the CV is observed at high scan rates (Figure 6b). This deformation is the consequence of a resistance increase due to some degradation occurring at this extended potential. HPOM seems to stabilize the system: the cathodic ESW, in the presence of HPOM, is slightly extended, as shown in Figure 3, and this explains the better CV shape retention at a high scan rate.

**Figure 7:**
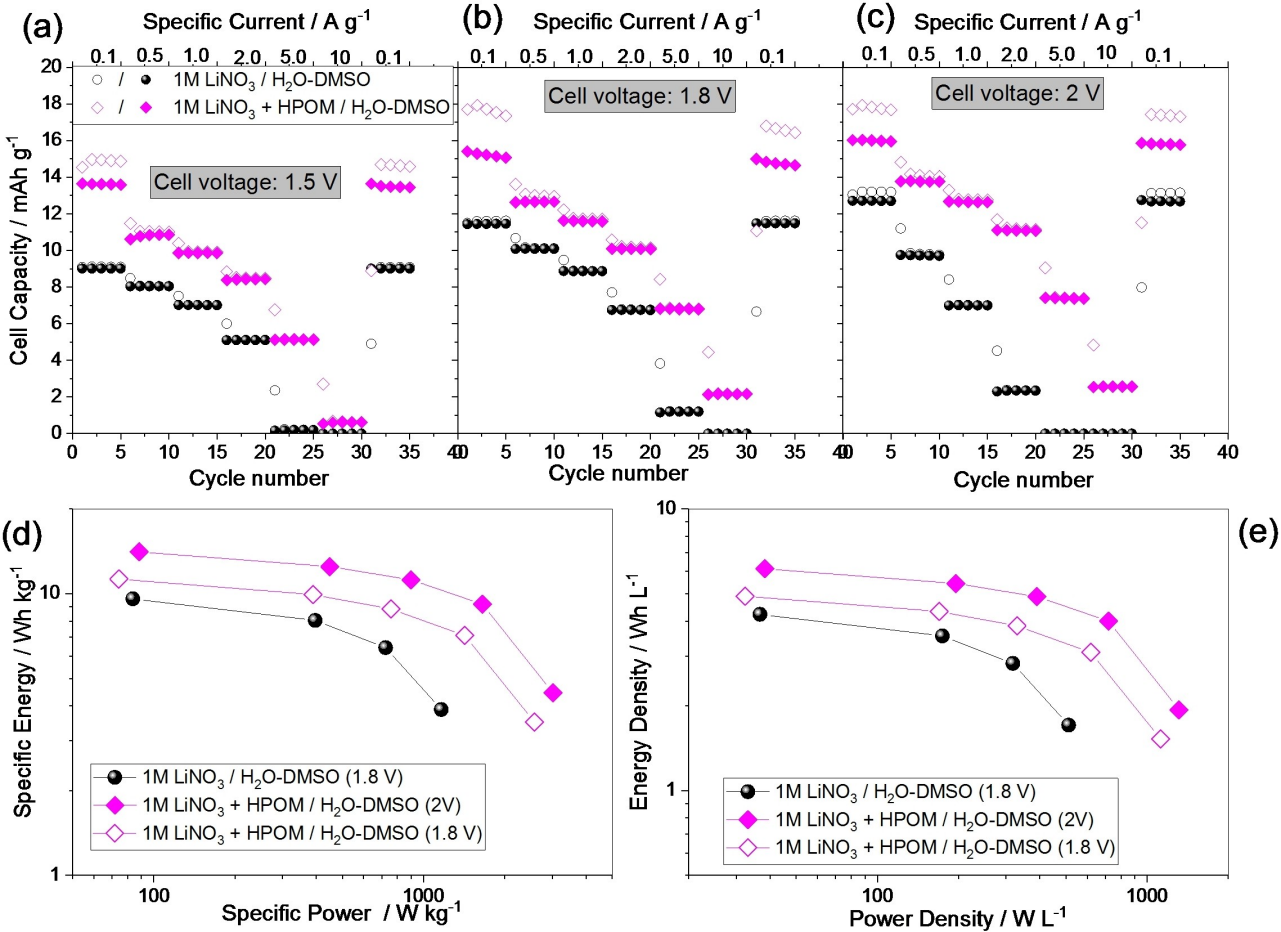
Galvanostatic charge discharge at increasing currents on AC//AC capacitors (ratio 2 : 1 positive: negative electrode) with selected electrolytes in the cell voltage of a) 1.5 V, b) 1.8 V and c) 2 V. Gravimetric (d) and volumetric (e) Ragone plots calculated from the galvanostatic cycles at different currents.

**Figure 8:**
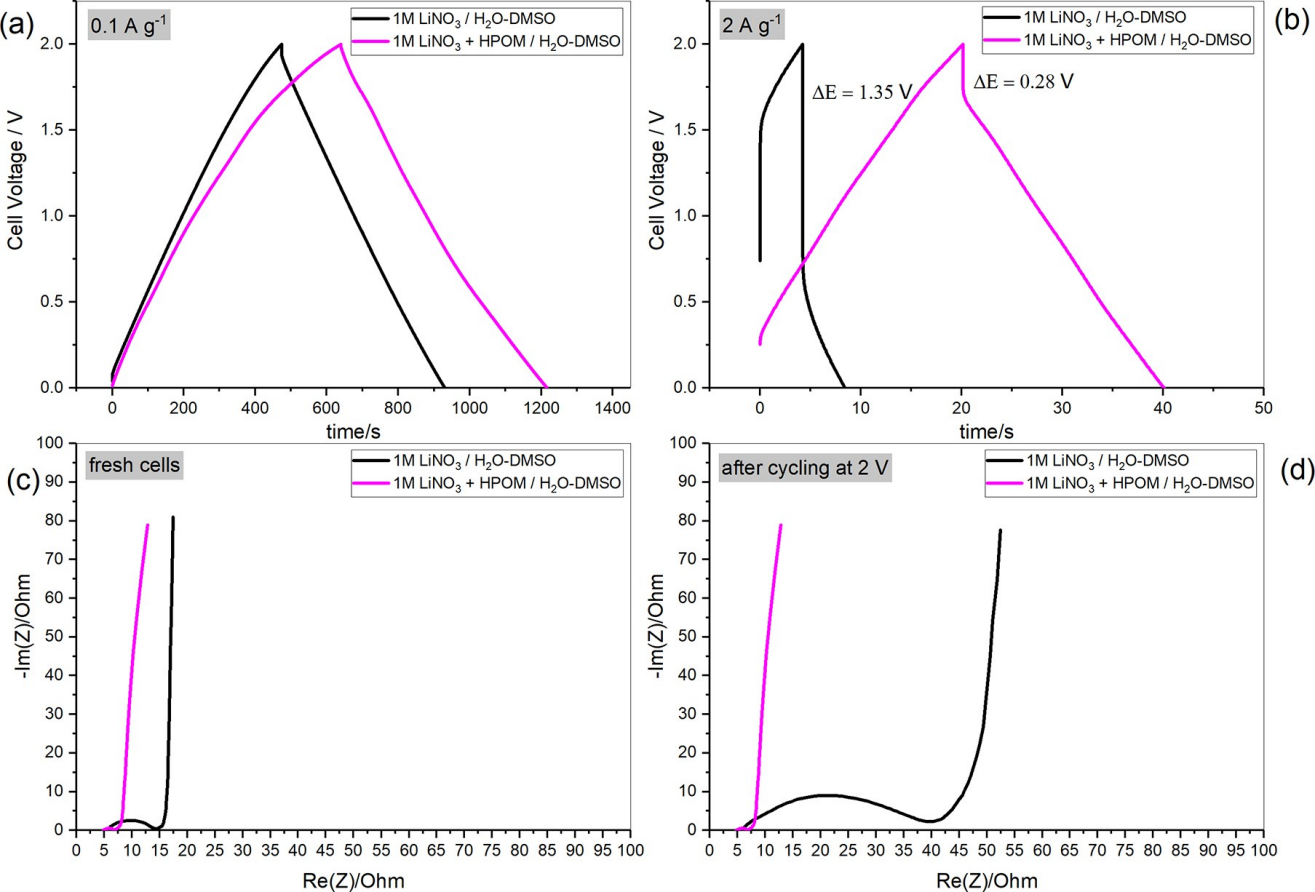
Galvanostatic profiles of AC//AC supercapacitors in the selected electrolytes recorded between 0–2 V at a) low current and b) high current; Nyquist plots of c) as‐assembled cells and d) cells subjected to the rate capability test at 2 V.

Due to the presence of redox peaks in the CV, in this work, the capacity, instead of the capacitance, is calculated and compared. Figure 7 shows the rate capability performance of the two cells cycled up to three different maximum voltages (1.5, 1.8, and 2 V). In every case, the capacity of the cell containing HPOM is higher. However, the presence of the redox species gives rise to a lower coulombic efficiency, especially at low currents. This drawback is expected when a faradaic reaction is involved. In the case of pure double‐layer charge storage, the efficiency is higher. One positive effect of the presence of the HPOM is the better rate capability and lower resistance. The difference in resistance is even more evident when the cells are cycled up to 2 V, as also highlighted by the iR‐drop (Figure 8b) and the EIS (Figure 8c).

The performance of the two devices is also transposed as Ragone plots, which shows the relation between energy accumulated and power. Figure 7c and d represents Ragone plots of the HPOM‐free cell cycled up to 1.8 V (the maximum reasonable cell voltage for the HPOM‐free electrolyte) and of the HPOM‐containing cell. As the HPOM‐containing cell can operate until 2 V, the performance at 1.8 and 2 V are reported. The Ragone plots highlight that the introduction of HPOM can increase the energy and power of the device. Further, the galvanostatic profiles in Figure 8 and S6, show a quasi‐linear potential variation with time in the presence of HPOM. The impedance of fresh electrodes is in the range of 5–15 ohm. The Nyquist plots (Figure 8c and d) show typical features as for supercapacitors: (i) a semicircle at high frequencies, indicating contact resistance between the current collector and the electrode material (with possible overlapping of charge‐transfer resistance in the case of redox electrolyte); (ii) a sloped region at middle‐to low frequencies, indicating the pores resistance; and a quasi‐vertical line at low frequencies, indicating the double layer capacitance.^32^ In agreement with the large iR‐drop of the HPOM‐free cell, developed during cycling at 2 V, the EIS spectra show a significant increase in the size of the semicircle. At the same time, the capacitive branch at low frequencies remains unchanged, indicating that the capacitance can still be accumulated, and the pores of the carbon are not blocked. The increase in the size of the semicircle can be attributed to a loss of contact among the particles and with the current collector.^32^ Degradation due to side reactions can also lead to the formation of a passivation layer (solid electrolyte interphase), which can contribute to the increase of the size of the semicircle.^39^ In the case of the cell containing HPOM, there is no relevant increase or change in the Nyquist plot.

**Figure 9:**
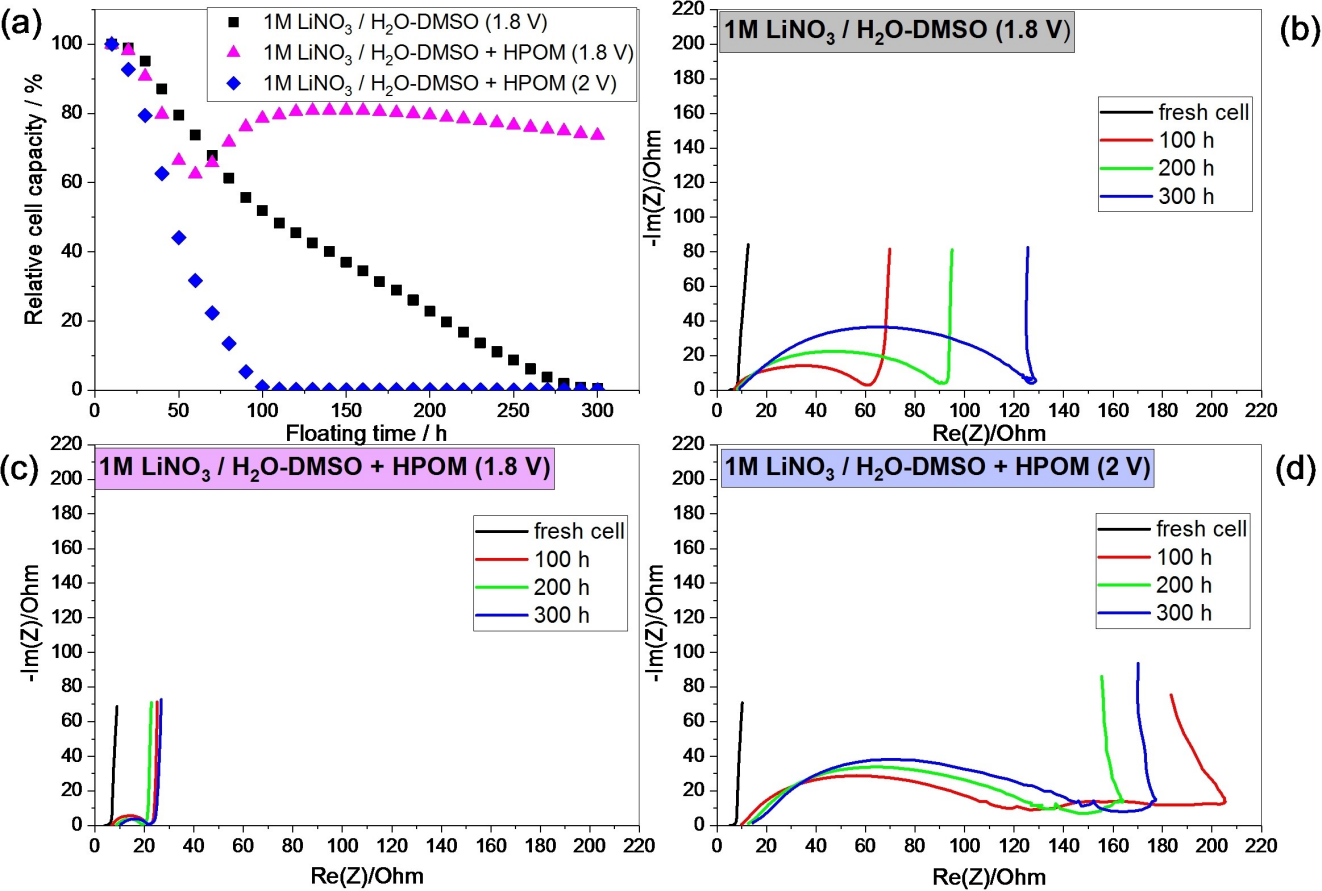
Ageing behavior under floating conditions of AC//AC supercapacitors (ratio positive: negative electrode 2 : 1). a) Capacity at 0.5 A g^−1^ versus floating time and evolution of the impedance spectra with the floating time of AC//AC capacitors in b) 1 M LiNO_3_/H_2_O‐DMSO electrolyte aged at 1.8 V, c) 1 M LiNO_3_+10 mM HPOM/H_2_O‐DMSO electrolyte aged at 1.8 V and d) 1 M LiNO_3_+10 mM HPOM/H_2_O‐DMSO electrolyte aged at 2 V.

However, it is crucial also to understand if these cells can operate for a long time and under stress conditions. Accelerated aging on supercapacitors can be performed under potential floating conditions. In this experiment, the cell is kept at the maximum voltage for a specific time, and the performance (*i. e*., EIS and GCPL) are periodically checked to monitor the aging level. This type of experiment is more indicative than to perform long‐term galvanostatic cycling because, in supercapacitors, the degradation reactions are more likely to occur at high potentials than under repeated cycling.^40^ Two cells based on the HPOM‐containing electrolytes are floated at 1.8 V and 2 V. For comparison, a cell HPOM‐free is floated at 1.8 V. Figure 9a reports the capacity retention versus floating time, as obtained from GCPL. Figures 9b, c, and d show the variation of EIS during floating. The capacity of the HPOM‐free cell continuously decreases during the floating time until reaching zero after 300 h of potential hold. Despite that the rate capability performance of the HPOM‐containing cell is not different for cell voltages of 1.8 and 2 V, relevant differences can be observed during the floating experiment. If the cell voltage is held at 2 V, the capacity rapidly decays, reaching zero after 100 h of floating. On the other side, the capacity of the cell floated at 1.8 V remains higher and constant until the end of the experiment. Nyquist plots, describing the increase of the cell impedance, are in line with the capacity retention. The impedance of the HPOM‐containing cell floated at 1.8 V remains at about 30 ohm, while the impedance of the other two cells rapidly grows, according to the capacity decay. The cell performance degradation can be easily explained by the hydrogen evolution at the negative AC electrode (as discussed earlier for Figure 4), which affects the aging of the AC/AC cells under floating conditions. The Figure S7, in the ESI file, shows that the potential of positive electrodes stays below 1.0 V vs. Ag/Ag^+^ up to a cell voltage of 2.0 V, meaning that the positive electrode works at almost constant potential during the floating period. On the other hand, the potential of the negative electrode is much lower for 1 M LiNO_3_/H_2_O‐DMSO than in 1 M LiNO_3_/H_2_O‐DMSO+HPOM at the same cell voltage of 1.8 V. In fact, the negative electrode demonstrates nearly constant potential profile throughout the floating period at 1.8 V. It only starts to show some variation when the cell voltage is increased to 2.0 V. At the voltage of 2.0 V, the negative AC electrode operates below −1.3 V vs. Ag/Ag^+^ which leads to the severe performance degradation^41^. This can be explained by the change in local pH at the negative electrode,^36^ becoming more alkaline, and degrading the POMs (Keggin‐type POMs are stable only at low pH^42^).

**Figure 10:**
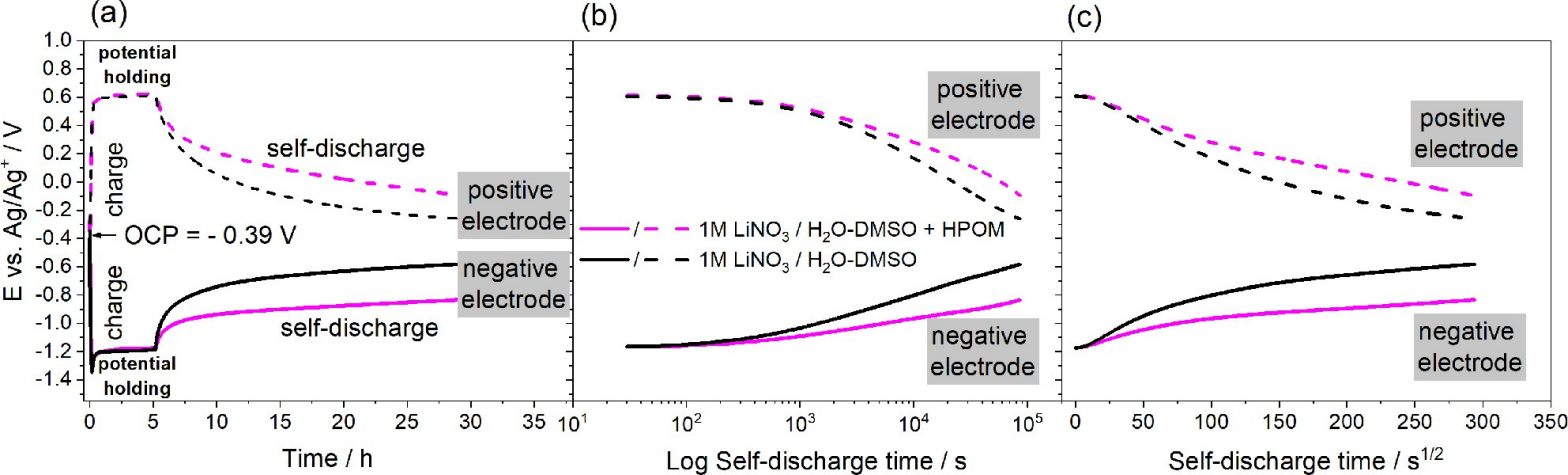
Self‐discharge profiles of a AC//AC capacitors (positive : negative electrode mass ratio 2 : 1) with 1 M LiNO_3_/H_2_O‐DMSO and 1 M LiNO_3_+10 mM H_3_PW_12_O_40_/H2O‐DMSO electrolytes. Potential changes of the positive electrodes (dashed lines) and the negative electrode (continuous lines) plotted as function of a) self‐discharge time, b) log self‐discharge time; and c) self‐discharge time^1/2^.

Self‐discharge of positive and negative electrodes in 1 M LiNO_3_/H_2_O‐DMSO+HPOM and 1 M LiNO_3_/H_2_O‐DMSO is estimated in a two‐electrode cell with a reference electrode and shown in Figure 10. Before starting the open circuit potential (OCP), a voltage hold of 5 hours at 1.8 V was applied for both systems. At this cell voltage, the negative electrode works at about −1.2 V vs. Ag/Ag^+^, which is a relatively safe potential range from the point of view of hydrogen evolution. The positive electrode potential is at about 0.6 V vs. Ag/Ag^+^, which is inside the stability limit depicted in Figure 3c. At the beginning of OCP, the potential of both positive and negative electrode decays exponentially (Figure 10a), however, for different reasons. While the negative electrode displays a potential decay of about 700 mV due to the strong shift of local pH from alkaline (provoked by OH^−^ generation from reduction of water) to neutral under the influence of bulk electrolyte pH,^43^ the positive electrode potential decay of 800 mV is due to the oxidation of electrode surface. The higher self‐discharge of the negative electrode in 1 M LiNO_3_/H_2_O‐DMSO is due to the hydrogen evolution (water reduction), which is stronger than in the cell with 1 M LiNO_3_/H_2_O‐DMSO+HPOM, where an improved self‐discharge is probably due to the strong adsorption of POMs species in carbon pores. The potential decay of negative and positive electrodes in the cell with 1 M LiNO_3_/H_2_O‐DMSO+HPOM is only 400 mV and 600 mV, respectively.

The potential versus log t curve in Figure 10b shows a similar trend in both systems up to 1000 s, which is followed by a more linear slope in 1 M LiNO_3_/H_2_O‐DMSO than in 1 M LiNO_3_/H_2_O‐DMSO+HPOM, indicating a more activation controlled process^43^ than in the former case at both positive and negative electrode. Figure 10c shows a potential versus *t* ^*1/2*^ curve, where a more linear trend is observed in the case of 1 M LiNO_3_/H_2_O‐DMSO+HPOM, indicating a predominant diffusion‐controlled process^44^ owing to the presence of adsorbed POMs at both positive and negative electrodes.

## 3 Conclusions

This work highlights the benefit of using DMSO‐H_2_O mixtures in supercapacitor devices. Due to the change of water structure, the hybrid organo‐aqueous system provides significant advantages, which can be relevant, not only for supercapacitors but also for other energy storage systems, like batteries. (1) The hydrogen evolution reaction is shifted to lower potentials, giving the possibility to expand the cathodic electrochemical stability window; (2) the corrosion of Aluminum is suppressed, which enables the use of this metal as a current collector. However, a drawback is that the presence of the organic solvent decreases the ionic conductivity.

Used together with a redox electrolyte based on POMs, other important properties of mixed DMSO‐H_2_O solvents is exploited: the presence of DMSO stabilizes the reduced form of phosphotungstic acid (H_3_PW_12_O_40_), and the redox reaction at low potentials becomes more reversible than in the pure aqueous system.

The AC/AC supercapacitor operated in 1 M LiNO_3_+10 mM H_3_PW_12_O_40_ in mixed DMSO‐H_2_O solvent displays improved performance in comparison to the analog POM‐free electrolyte. POMs increase the energy density due to the addition of multi‐steps redox reactions to the double layer charge storage. At the same time, the presence of POMs improves the stability of the cell under floating conditions at 1.8 V. The improved stability is explained in terms of hydrogen evolution. The hydrogen evolution is further shifted to lower potentials by the POM adsorbed on the AC electrode. The POMs modify the hydrogen evolution potential, giving rise to a more stable system. The lower self‐discharge behavior further confirms the strong adsorption of POM moieties in the AC pores in comparison to a POM‐free cell.

In this work, only one commercial activated carbon, and only one type of POMs were combined as proof of concept. Further work should be done on the matching of activated carbon with tuned pores and functionalities with various types of polyoxometalates. Moreover, in‐situ techniques, such as in‐situ Electrochemical Quartz Microbalance (eQCM) and in‐situ UV‐VIS spectroscopy can help to further understanding the electrochemical mechanism occurring during charge and discharge at the interface electrode‐electrolyte.

Finally, the physical properties of the hybrid aqueous/non‐aqueous system inherit the merits from both aqueous (non‐flammability, high conductivity) and non‐aqueous (high electrochemical stability, low freezing point) systems. Since the system DMSO‐H_2_O benefits of a low freezing point, future work will be addressed to the characterization of supercapacitor devices at low temperatures. The exploration of other organic solvent to prepare binary organic‐aqueous electrolyte can be of interest for supercapacitor‐ and battery‐related communities.

## Experimental Section

### Electrolytes

LiNO_3_, H_3_PW_12_O_40_, 1,4**‐**Dioxane (DO) and Dimethyl sulfoxide (DMSO) were purchased from Sigma Aldrich.

H_3_PW_12_O_40_ was dried at 50 °C under vacuum for 24 hours in order to remove the adsorbed water.

The electrolytes used in this work are listed below:

1. 1 M LiNO_3_ in H_2_O
2. 1 M LiNO_3_ in H_2_O: DMSO (1 : 1 v/v)
3. 1 M LiNO_3_ in H_2_O: DO (1 : 1 v/v)
4. 1 M LiNO_3_+1 mM H_3_PW_12_O_40_ in H_2_O
5. 1 M LiNO_3_+1 mM H_3_PW_12_O_40_ in H_2_O: DMSO (1 : 1 v/v)
6. 1 M LiNO_3_+1 mM H_3_PW_12_O_40_ in H_2_O: DO (1 : 1 v/v)
7. 1 M LiNO_3_+10 mM H_3_PW_12_O_40_ in H_2_O
8. 1 M LiNO_3_+10 mM H_3_PW_12_O_40_ in H_2_O: DMSO (1 : 1 v/v)

The conductivity of the electrolytes was measured with a Mettler Toledo conductometer at 23 °C. The pH was measured with a Mettler Toledo (Five Easy f20) pH meter at 23 °C.

### Glass‐Cell Set‐Up

The electrochemical study of the electrolytes was done in a glass cell with Teflon cup. A glassy carbon (area: 0.071 cm^2^) was used as the working electrode and polished with Al_2_O_3_ suspension before each experiment. Pt wire was used as the counter electrode and Ag/AgCl as the reference electrode. For the corrosion experiment, Al foil was used as the working electrode. Before each experiment, the electrolyte was purged with N_2_ flow.

### Activated Carbon Electrode Preparation and Cell Assembly

A commercial activated carbon (Haycarb PLC), which was extensively characterized in our previous works^45^ was employed as the active material for both positive and negative electrodes. This activated carbon has a BET surface area of 1705 m^2^ g^−1^ and a meso‐ and micropore volumes of 0.281 and 0.646 m^3^ g^−1^, respectively. The average pore diameter is 1.2 nm. The working electrodes were prepared by mixing activated carbon as active material and SuperP (Imerys) as conductive additive with a binder solution in the following way: (i) PVDF binder (Solvay) was firstly dissolved in NMP (Sigma Aldrich), (ii) activated carbon and SuperP were then successively added to the binder solution. The final slurries were coated on Al foil with wet thicknesses of 170, 200, 300 and 350 μm. The variation of the thickness allowed us to select appropriate electrodes for the desired mass ratio. The cast layers were dried first for 2 h at 60 °C and then at 80 °C for 10 h to remove any residual solvent. Circular electrodes (with 12 mm diameter) were punched out from the layer and pressed at 7 tons with a hydraulic laboratory press.

Swagelok® type cells have been used for the electrochemical experiments in the full cell. AC electrodes were used as positive and negative electrodes in a mass ratio of 2 : 1. An oxidized silver was used as a reference electrode. Positive and negative electrodes were separated by two glass microfiber filter separators (Whatman® GF/A, Aldrich). 450 μl of the electrolyte solution was injected into the cell.

Swagelok® type cells were also used for half‐cell configuration to determine the electrochemical stability window and the hydrogen storage ability. In this case, the configuration was the same as in the full cell, but an oversized AC electrode (10 times heavier electrode) was used as the counter electrode.

### Electrochemical Measurements

- Electrochemical experiments in glass cell configuration
– cyclic voltammetry on glassy carbon were recorded with a scan rate of 10 mV s^−1^.
– The corrosion test on the Al foil was conducted at 1 mV s^−1^.
- Electrochemical experiments in Swagelok®‐type cells (full cells)
– Cyclic voltammetry (CV), Potentiostatic Electrochemical Impedance Spectroscopy (PEIS) and Galvanostatic cycling with potential limitation (GCPL) were performed on full cells sequentially by using the following protocol:
- CV curves were recorded at 5 and 10 mV s^−1^. GCPL was performed at currents ranging from 0.1 to 10 A g^−1^. In the specific current calculation, the sum of the mass of positive and negative electrode active materials was considered. The cells were cycled in three different voltage ranges (0–1.5 V, 0–1.8 V, and 0–2 V). Potentiostatic electrochemical impedance spectroscopy (PEIS) was performed at the bias cell voltage of 0 V in the frequency range 500 kHz–10 mHz and with a sinus amplitude of 5 mV.
– The aging floating experiment was performed by holding the cell voltage at 1.8 V or 2 V for a total of 300 hours. Every 10 hours of floating, the performance of the cell was evaluated by PEIS, and five galvanostatic cycles at 0.5 A g^−1^.
– Self‐discharge experiment: Before measuring self‐discharge, the cell was charged to 1.8 V with 0.1 A g^−1^, and the voltage was held at that value for 5 hours. Afterward, the voltage drop (and as well as the potential drop of individual electrodes) was recorded at the open circuit for 24 h.
- Electrochemical experiments in Swagelok®‐type cells (half cells)
– Electrochemical stability window (ESW)
– ESW was determined with cyclic voltammetry measurements (CV) performed at 1 mv s^−1^. In detail, starting from the open‐circuit voltage (OCV), the potential was increased in the negative or positive direction with 0.1 V steps until the final potentials of −1.6 V and +1.6 V vs. Ag/Ag^+^, respectively.

### Hydrogen storage experiment

The cyclic voltammograms (CVs) were performed at 2 mV s^−1^, and the lower potential limit was step‐wise decreased until −1.6 V vs. Ag/Ag^+^. The positive potential cut‐off was 0.6 V. Each CV is collected after performing three cycles at a given potential limit in order to ensure proper equilibration of the system.

All electrochemical tests were conducted on a VMP3 Potentiostat (BioLogic) equipped with EC‐Lab software.

## Conflict of interest

The authors declare no conflict of interest.

## Supporting information

As a service to our authors and readers, this journal provides supporting information supplied by the authors. Such materials are peer reviewed and may be re‐organized for online delivery, but are not copy‐edited or typeset. Technical support issues arising from supporting information (other than missing files) should be addressed to the authors.

Supplementary
