# Supplementary material for: Benefits of Organo‐Aqueous Binary Solvents for Redox Supercapacitors Based on Polyoxometalates
Source: ChemElectroChem. 2020 Jun 10;7(11):2466–76. doi: 10.1002/celc.202000639 (PMC7319425; doi:10.1002/celc.202000639)
Supplement: Supplementary file 1 — Supplementary [file CELC-7-2466-s001.pdf]

ChemElectroChem

Supporting Information

Benefits of Organo-Aqueous Binary Solvents for Redox Supercapacitors Based on Polyoxometalates

Sonia Dsoke* and Qamar Abbas© 2020 The Authors. Published by Wiley-VCH Verlag GmbH & Co. KGaA. This is an open access article under the terms of the Creative Commons Attribution License, which permits use, distribution and reproduction in any medium, provided the original work is properly cited.

Electronic Supplementary Information

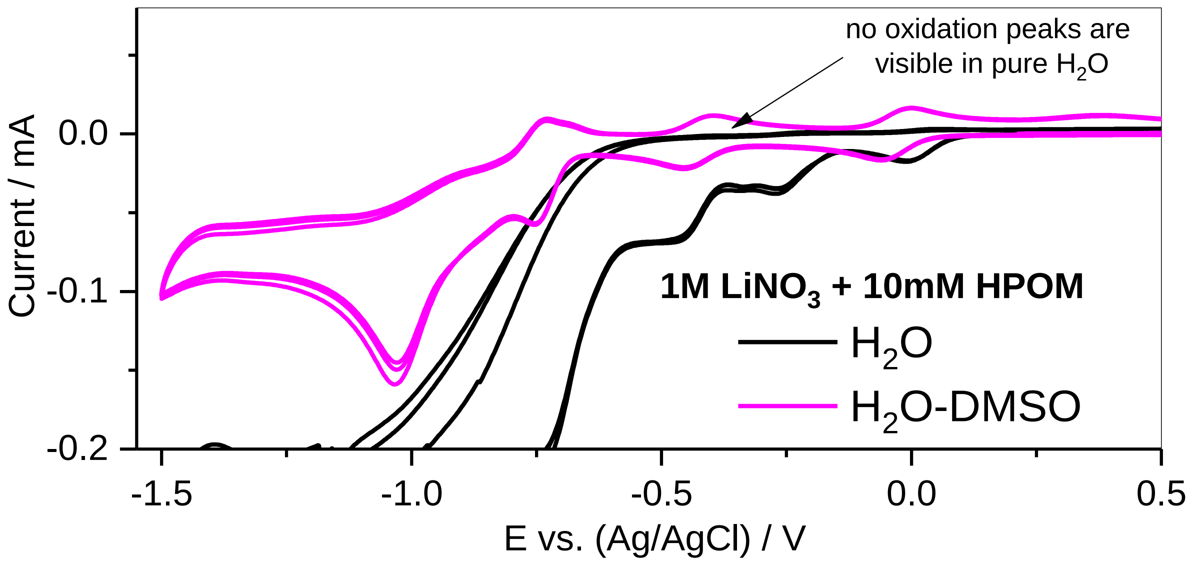

S1 - Zoom of Figure 1 c of the Manuscript: The presence of DMSO increases the reversibility of the redox reactions. No oxidation peaks can be observed in H2O-based elctrolyte (black curve).

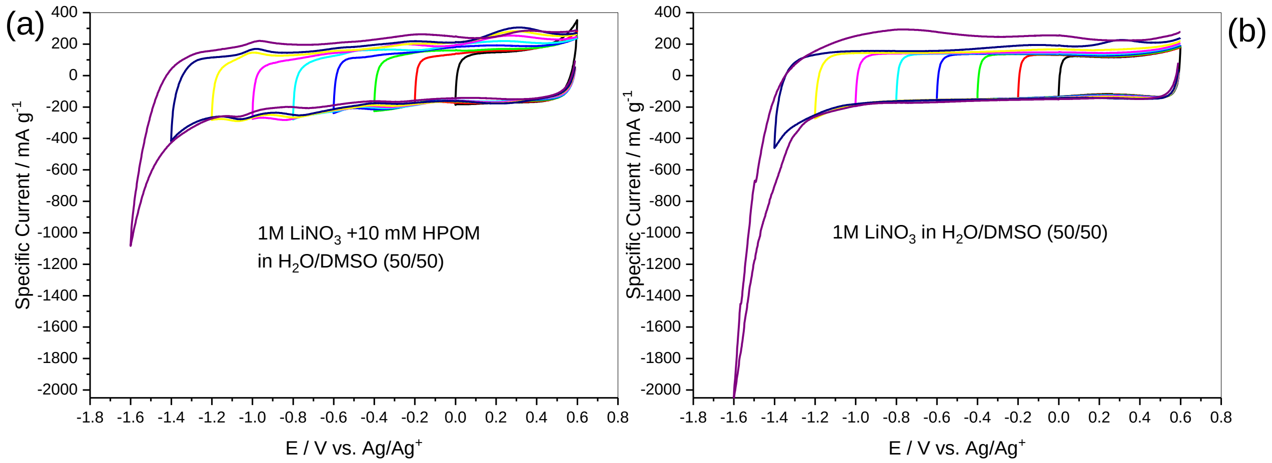

S2 - Cyclic voltammograms in three-electrode setup on AC working electrode at 2 mV s^{-1} in 1M $LiNO_3/H_2O$-DMSO+HPOM (a) and 1M $LiNO_3/H_2O$-DMSO (b).

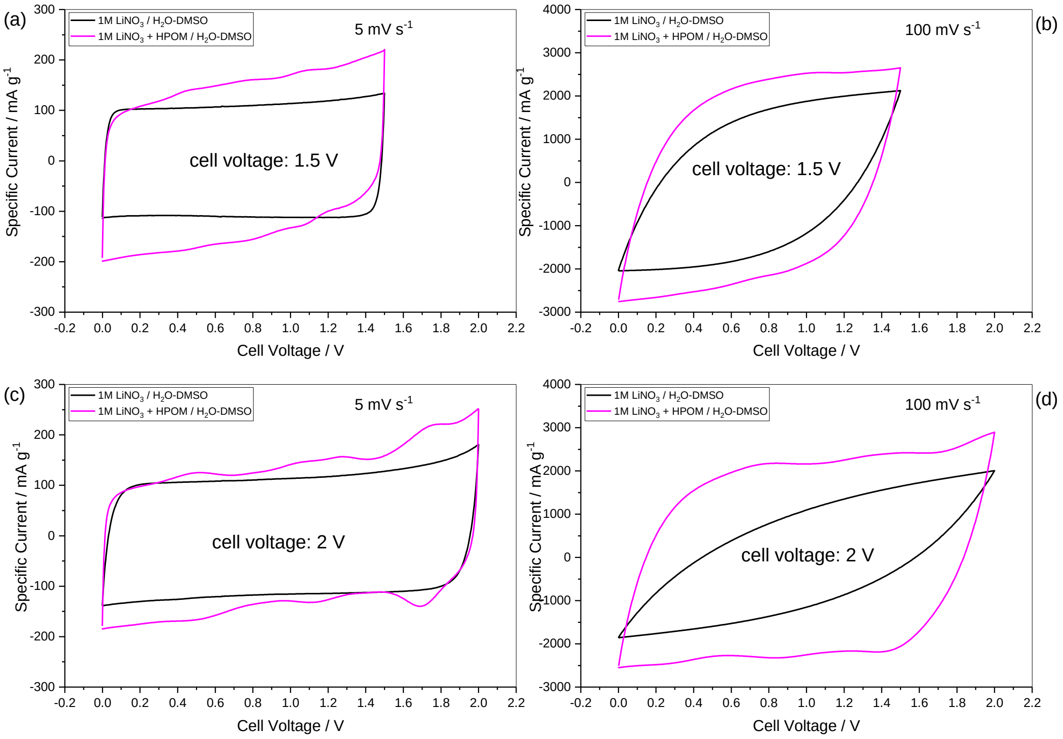

S3 Cyclic voltammetry profiles at 5 mV s^{-1} and 100 mV s^{-1} of AC//AC supercapacitors with a mass ratio of 2:1 (positive:negative electrode) and selected electrolytes in the cell voltage of 1.5 V (a and b) and 2 V (c and d).

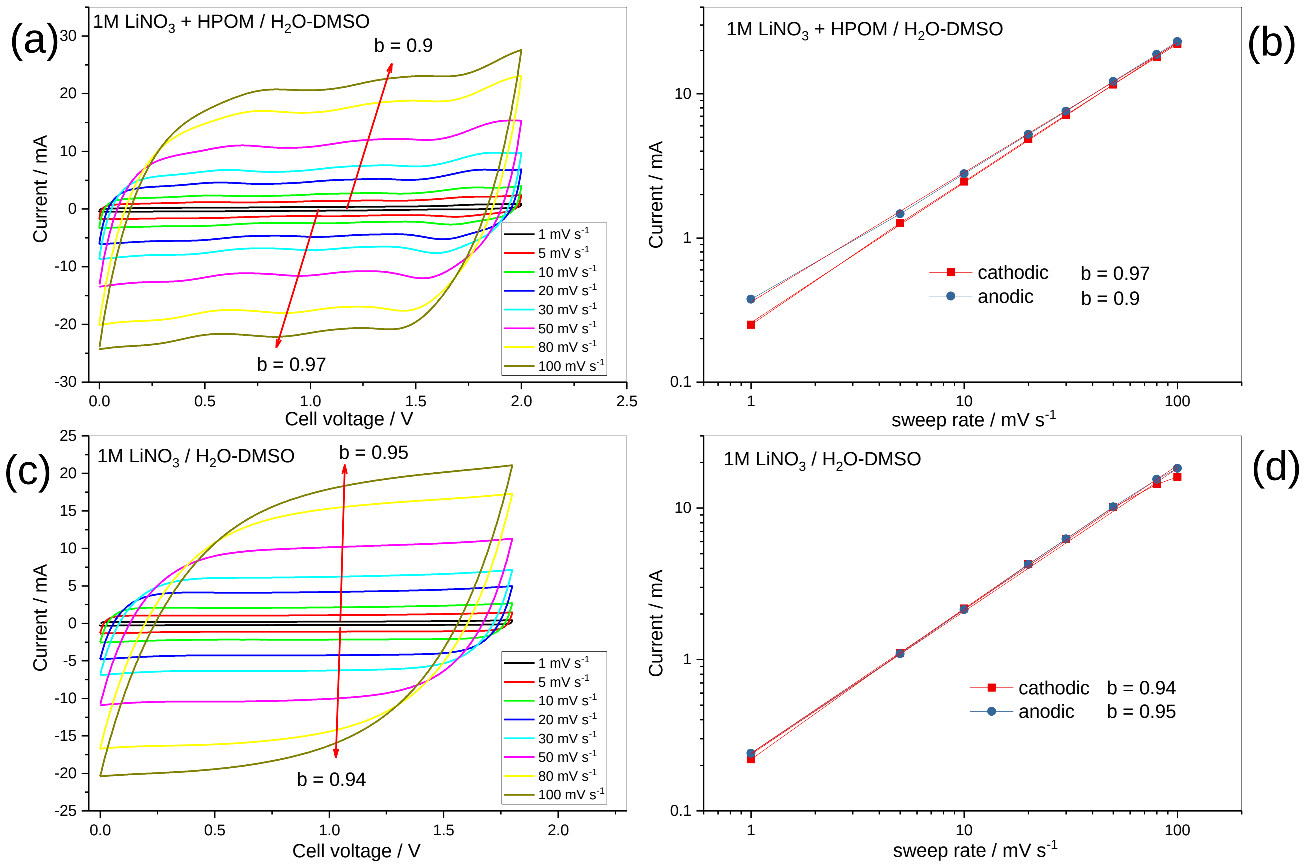

S4 Cyclic voltammetry at increasing scan rates (5 up to 100 mV/sec) of AC//AC supercapacitors with a mass ratio of 2:1 (positive:negative electrode) and selected electrolytes (a and c). Linear fit of the peak current (for the HPOM-containing electrolyte) and of the current at 1 V (for the HPOM-free electrolyte).

Calculations:

The peak current is in relation with the scan rate by following the power law[1]:

$$i(v) = a\cdot v^{b} \qquad \text{(Eq.1)}$$

$$\text{Log } i(v) = \text{Log a} + \text{b Log } v \qquad \text{(Eq. 2)}$$

where both, a and b, are adjustable parameters.

The parameter b is calculated from the slope of the linear plot of log *i* vs. log *v* and is used to provide kinetic information about the electrochemical process.

If the slope b =1, the electrochemical reactions occurs through a surface-controlled process (i.e. capacitive-like). If the slope b = 0.5, the system is dominated by diffusion-controlled processes.

In S4, it is clear that in both presence or absence of POMs the reaction occurs predominantly via a surface charge storage.

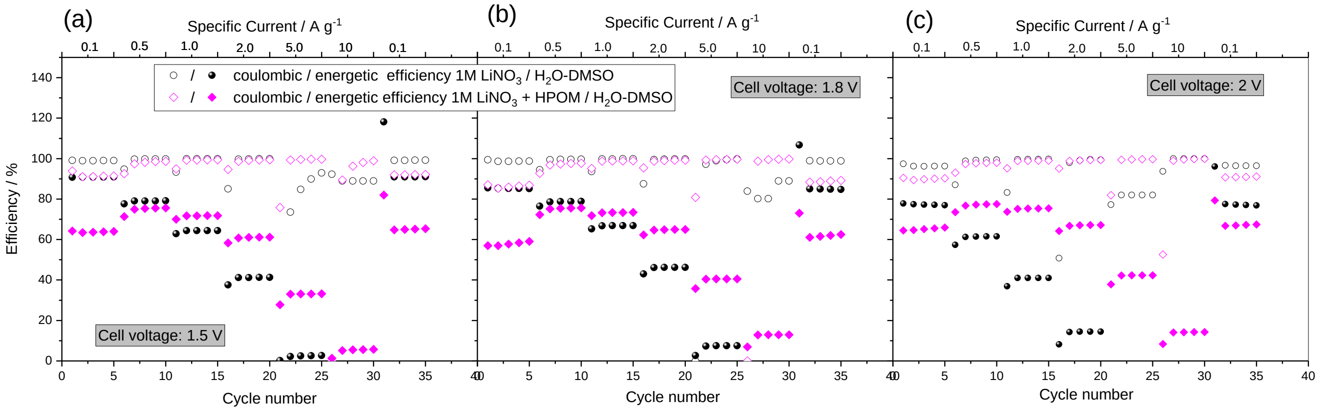

S5 - Coulombic and energetic efficiency during the rate capability test.

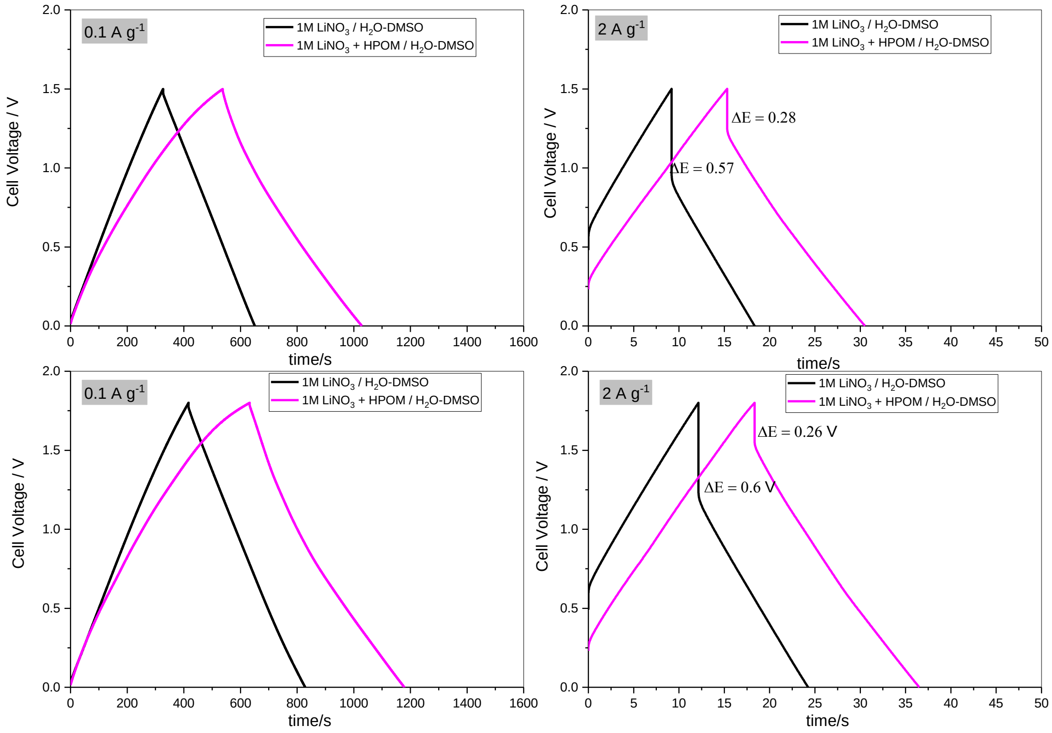

S6 – Galvanostatic profiles at 0.1 and 2 V in the cell voltage of 1.5 and 1.8 V

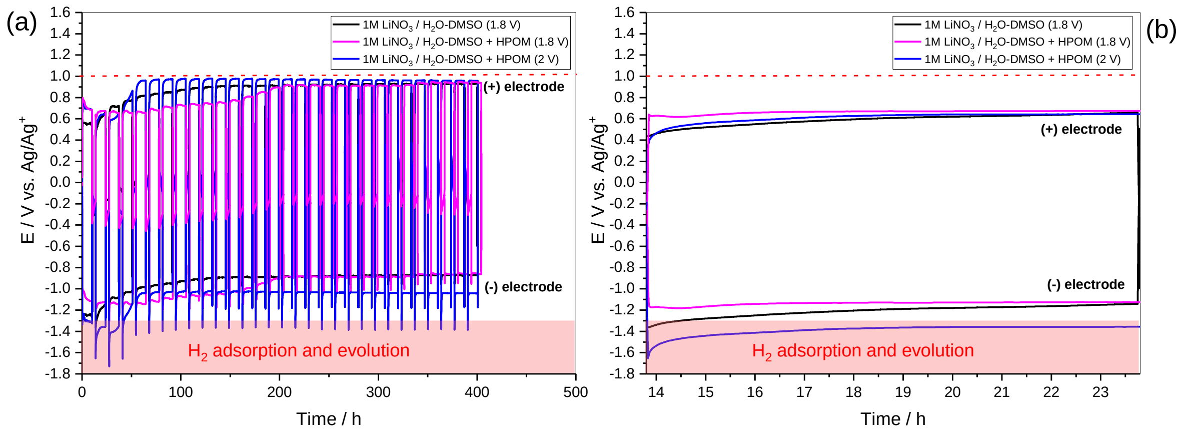

S7 - Potential profiles of positive and negative electrodes during potentiostatic floating of AC/AC cells in 1M $LiNO_3/H_2O$-DMSO at 1.8 V (black curve), in 1M $LiNO_3/H_2O$-DMSO+HPOM at 1.8 V (green curve) and in 1M $LiNO_3/H_2O$-DMSO+HPOM at 2.0 V (blue curve). The light red area indicates the hydrogen evolution potential region and the upper dashed line indicates the water oxidation potential limit.

[1] J. Liu, J. Wang, C. Xu, H. Jiang, C. Li, L. Zhang, J. Lin, Z. X. Shen, *Advanced science (Weinheim, Baden-Wurttemberg, Germany)* **2018**, *5*, 1700322.
